# Global Patterns of Guild Composition and Functional Diversity of Spiders

**DOI:** 10.1371/journal.pone.0021710

**Published:** 2011-06-29

**Authors:** Pedro Cardoso, Stano Pekár, Rudy Jocqué, Jonathan A. Coddington

**Affiliations:** 1 Smithsonian Institution, National Museum of Natural History, Washington DC, United States of America; 2 Azorean Biodiversity Group - CITA-A, Universidade dos Açores, Angra do Heroísmo, Portugal; 3 Department of Botany and Zoology, Faculty of Science, Masaryk University, Brno, Czech Republic; 4 Musée Royal de l'Afrique Centrale, Tervuren, Belgium; University of Pretoria, South Africa

## Abstract

The objectives of this work are: (1) to define spider guilds for all extant families worldwide; (2) test if guilds defined at family level are good surrogates of species guilds; (3) compare the taxonomic and guild composition of spider assemblages from different parts of the world; (4) compare the taxonomic and functional diversity of spider assemblages and; (5) relate functional diversity with habitat structure. Data on foraging strategy, prey range, vertical stratification and circadian activity was collected for 108 families. Spider guilds were defined by hierarchical clustering. We searched for inconsistencies between family guild placement and the known guild of each species. Richness and abundance per guild before and after correcting guild placement were compared, as were the proportions of each guild and family between all possible pairs of sites. Functional diversity per site was calculated based on hierarchical clustering. Eight guilds were discriminated: (1) sensing, (2) sheet, (3) space, and (4) orb web weavers; (5) specialists; (6) ambush, (7) ground, and (8) other hunters. Sixteen percent of the species richness corresponding to 11% of all captured individuals was incorrectly attributed to a guild by family surrogacy; however, the correlation of uncorrected vs. corrected guilds was invariably high. The correlation of guild richness or abundances was generally higher than the correlation of family richness or abundances. Functional diversity was not always higher in the tropics than in temperate regions. Families may potentially serve as ecological surrogates for species. Different families may present similar roles in the ecosystems, with replacement of some taxa by other within the same guild. Spiders in tropical regions seem to have higher redundancy of functional roles and/or finer resource partitioning than in temperate regions. Although species and family diversity were higher in the tropics, functional diversity seems to be also influenced by altitude and habitat structure.

## Introduction

Hutchinson [1] was the first to suggest that species were limited to ecological niche boundaries by competing species [2]. Groups of competitors, or “a group of species that exploit the same class of environmental resources in a similar way”, were later called guilds by Root [3], [4]. In the meantime, many different definitions of guilds were used, in a relatively loose way [5], [6]. The currently most accepted definition characterizes ecological guilds as non-phylogenetic groups of species that share one or a series of important resources [7].

Parallel to guilds, functional groups were defined as groups of species that have the same function in the ecosystem, providing the same ecosystem services. Although guilds and functional groups are different concepts, with the first focusing on resource sharing and the latter focusing on ecosystem processes, the groups formed by both approaches often overlap [4], [7], [8]. Guild members may have similar functional roles in the communities, in which case both terms define the same set [7].

The definition and study of guilds is especially useful if they respond in roughly the same way to similar changes in the environment, independently of the specific taxonomic composition. Studying ecological guilds (or functional groups) can be useful to investigate assemblage response to climate change [8]–[10], habitat disturbance [11], [12], management [13] among many other areas [6].

The study of guild structure implies its quantification. Functional diversity is one of the most important parameters used to explain how ecosystems work and adapt to change [14], [15]. In order to quantify guild and functional group diversity, a number of complex and precise measures have been developed during the latter decade. Using total dendrogram branch length to measure functional diversity was first proposed by Petchey, Gaston [16] as a more useful measure than simply counting the number of guilds or functional groups [14].

Higher trophic levels have been repeatedly found to be especially sensitive to environmental change, either because they operate at a larger spatial scale than other groups, becoming more sensitive to, e.g., fragmentation, or because they are subject to the same factors as lower trophic levels as well as being strongly dependent on lower trophic groups and their changes, thus experiencing a synergistic effect [8]. Spiders therefore ought to be a good indicator taxon to reflect ecological change. They are in fact the main arthropod predators in many biomes and habitat types. Additionally, they have already been suggested to be an ideal group for predicting extinction debt in other taxa due to habitat destruction [17]. Classifying spiders into guilds seems therefore useful to future studies of ecological change in all kinds of biomes and habitats.

Several authors have tried to define spider guilds by using foraging strategies to predict arthropod prey group as the shared resource [18], [19]. Flying arthropods are mainly captured by different types of webs, epigean arthropods by wandering spiders or tube web hunters, arboreal arthropods by sheet webs, etc. Therefore, although many guild classification systems exist for spiders, these are usually based solely on foraging strategy, although different strategies may be directed towards similar prey and similar strategies may be directed towards different prey.

Based on comprehensive spider inventories of a number of forest sites in different parts of the world, our first objective was to define spider guilds that can be applied to all extant families worldwide. Comparing spider assemblages in different regions is possible if guild and functional diversity patterns are global, even if taxonomic composition is disparate [19]. Because spiders are among the most abundant and diverse predators in all kinds of terrestrial biomes worldwide and because predators are predicted to be especially sensitive to ecological change, it is important to define guilds applicable at a worldwide level and to verify if guilds and functional diversity are potentially useful to make comparisons among taxonomically disparate assemblages. We certainly realize that the current scope of spider families as presently defined in some cases is so broad that one family may include various guilds, functional groups and foraging strategies, but it nevertheless seems worthwhile to investigate the possibility and to assess critically its success. This is the first time such a goal is attempted at a global scale for spiders and, to our knowledge, for any invertebrate group.

The second objective was to test the hypothesis that guilds defined at family level are good ecological surrogates of guilds defined at the species level. Guild classification should ideally be made at the species level, because each species usually has a uniform behavior, which may be different from any other, even closely related, species [19]. However, it is impossible to assign guilds to all spider species, or even genera. Currently 110 families (two new families have recently been recognized), 3,821 genera, and more than 42,000 spider species are known [20]. Rates of description are high, limited mainly by the taxonomists available, and based overwhelmingly on museum specimens without ecological data. The behavior of probably 90% of the described species is unknown and, if necessary, inferred from the genus or family to which they belong. Phylogeny is probably the best predictor of ecology, but even at the family level spider phylogeny is not robustly known. Intrafamilial phylogenies are scarce, equivocal, and almost universally based on dramatically incomplete samples. About 75% of the genera contain five or fewer species and many are known only from the original literature description. Roughly half of the species descriptions are pre-1940 and contain only morphological information. Nevertheless, experienced araneologists expect to identify animals in the field to family from behavior, habits, habitat, and appearance, so “ecology” is relatively predictable at coarse scale. Therefore, using higher taxa surrogates at the family level may be as justified for guild classification and functional diversity quantification (ecological surrogacy) as it is for taxonomic diversity quantification (taxonomic surrogacy) [21]. Given that family members tend to have similar lifestyles [18], [22], [23], we hypothesize that using family surrogates can be an appropriate strategy.

The third objective was to compare the taxonomic (family) and guild composition of spider assemblages from different parts of the world. Taxonomic composition of assemblages varies wildly between sites in different biomes. Usually no native species and only a few genera are shared between temperate and tropical forests. Families may be exclusive to particular regions; richness or abundance of families usually differ. However, spiders and their role as one of the main predator taxa in all terrestrial ecosystems are ubiquitous. If ecosystem services are similar worldwide, with all ecosystems needing the same functional components independently of what taxa perform which tasks, different communities may present similar guilds in similar proportions even if the taxonomic composition differs. This similarity of guilds in different regions forms the basis for the apparent convergence of distant assemblages [5], [24]. We therefore hypothesize that guild composition is more stable, i.e. constant in proportions, at global scales than taxonomic composition.

Our fourth objective was to compare the taxonomic and functional diversity of spider assemblages from different parts of the world. Tropical regions are known to be major hotspots of biodiversity, with species richness reaching its peak for most animal taxa. A number of alternative or complementary hypotheses have been suggested to explain this almost universal pattern, from tropical climates being older and historically larger, allowing more opportunity for diversification [25] to geometric constraints on species richness [26], [27]. However, higher richness may not equate to higher functional diversity, because species may have partly redundant roles and/or establish a finer resource partitioning. Given that co-occurring taxonomically similar species tend to diverge in their functional roles in order to avoid competition, poorer assemblages may present species that occupy the available niches as thoroughly as the species in richer assemblages. We hypothesize that although taxonomic (family) richness is considerably higher in the tropics, differences in functional diversity will be much less, at least between different biomes, and will show higher redundancy and/or finer resource partitioning in lower latitudes.

Our fifth and final objective was to relate functional diversity with habitat structure and complexity. If functional diversity remains mostly similar across latitudes and biomes, other explanations must be sought to understand differences between sites. Habitat structure may be responsible for such differences. A site with major vegetation complexity can present more variety of prey or simply more opportunities for spiders to build snares and retreats. We hypothesize that functional diversity is positively related with habitat complexity, with more complex habitats being more functionally diverse.

## Materials and Methods

### Study sites and sampling procedures

This study is based on datasets from work on spider diversity over the last twenty years under diverse conditions and objectives. Seven forest sites were chosen in different regions of the world to provide a reasonable synopsis of global spider diversity (Table 1). Sampling followed the semi-quantitative design of Coddington et al. [28], with different effort per method at each site (see references in Table 1). In this kind of sampling, each sample represented one method applied for 1 h of active, continuous collecting (i.e. including time required to transfer the specimens to a vial, but excluding interruptions). Semi-quantitative sampling as applied for this work, especially if optimized, was recently found to be extremely efficient and capable of guaranteeing maximum richness with minimum effort but still allowing comparability of sites by using a standard set of methods and effort per method and time of day [34], [35]. Sampling was made by different teams at each site, however, all teams had a mix of experienced and inexperienced collectors and collecting experience with semi-quantitative sampling was previously found to be relatively less important than method and time of day for sampling efficiency [30], [31], [36]. We limited the sites to forests, from temperate to tropical, to reduce variance. Savannah or similar open habitats require a different set of sampling methods [34], [35], [37]. In Cameroon we had to lump data from two different plots in order to increase sample number, however, 84% of the data came from a single plot. The higher elevation locales in the tropics had lower canopies and in general a simpler habitat structure than low elevation sites.

**Table 1:** Overview of studied sites and respective biogeographical, ecological and spider assemblage characteristics.

Site	Climatic region	Habitat type	Altitude (m)	Family richness	Species richness	Number of individuals	Estimated species richness (Chao 1)	Reference
USA	Temperate	Mesic hardwood forest	800	17	76	1228	108	[29]
Portugal (Gerês)	Temperate/Mediterranean	Mixed English oak (*Quercus robur* L.) and Pyrenean oak (*Quercus pyrenaica* Willd.) woodland	650	25	117	1795	142	[30]
Portugal (Arrábida)	Mediterranean	Cork oak (*Quercus suber* L.) woodland	60	26	93	1473	106	[31]
Guyana	Tropical	Lowland moist forest	240	31	228	2934	234	[32]
Cameroon	Tropical	Mid-elevation moist forest	800	32	218	1407	310	J.A. Coddington et al., unpublished data
Tanzania	Tropical	High-elevation moist forest	1850	31	120	2330	155	[33]
Madagascar	Tropical	Mid-elevation moist forest	1000	33	291	3167	334	J.A. Coddington et al., unpublished data

Because for every site we had a different number of samples per method, to guarantee the comparability of datasets for each one, we considered 32 samples of each of three methods:

Aerial searching - Hand collection with pooter, vial, forceps or brush from knee level to as high as the collector could reach.

Beating - Branches of trees and other vegetation were beated with a wooden stick while holding a 1-m square beating tray underneath to catch the falling specimens.

Ground searching - Hand collection from ground level to knee height.

All three methods were applied equally during day and night (spiders are mainly nocturnal), i.e., 16 hours of sampling per method/time of day in each site. In the few cases when enough samples of one method/time of day combination were not available (diurnal aerial sampling in Guyana (12 samples) and nocturnal beating in Cameroon (9 samples)) the missing data were substituted by samples of the same method but a different time.

### Ecological data

The definition of guilds should be based on ecological characteristics of species (or higher taxa) that determine resource sharing. As mostly generalist predators of arthropods, the most important resource for spiders is arthropod prey, and their most important distinctive characteristics probably are their foraging method, the range of prey they hunt, vertical stratification, circadian activity, body size and phenology. Body size and phenology within spider families are extremely variable worldwide, and present wide disparities in these traits. Given the broad scale and exploratory character of this study, we therefore have not considered such traits, although they are no doubt important in structuring assemblages at a local scale. In this work we used information on foraging strategy (type of web and method of active hunting), prey range (either stenophagous or euryphagous), vertical stratification (ground or vegetation) and circadian activity (diurnal or nocturnal) (Table S1).

Data for each family was collected from a number of sources. We used the general characteristics of families [18], [22], [23], acknowledging that exceptions in many cases are inevitable at such a large taxonomic and geographic scale. In a few families in which relatively large numbers of species clearly have evolved distinct lifestyles (i.e. not just the exception), we separated the families into sub-families and classified each accordingly. These sub-families are hereafter treated as families. When families were largely unknown, we used the characteristics of a particular species in the family for which the behavior was known. Additionally, we analyzed data from a number of exhaustive samples combining methods targeted towards different vertical strata and times of day [28]–[33], [36], [37]. Such data allowed inferring on the vertical stratification and circadian activity of families. All characteristics were evaluated only in a binary way (Table S1).

### Definition of guilds

The definition of guilds can be made *a priori*, based on certain characteristics thought to be especially important [8], [38]–[40], or *a posteriori*, using quantitative methods to find natural groups [41]. The latter reduces the subjectivity of guild placement. However, resulting groups may seem less natural to the experienced researcher [42]. Among quantitative methods for the definition of guilds, we may include nearest-neighbor variance in overlap [43], multivariate analysis [44], clustering algorithms [18], [45], [46], psychometric analysis [47] and bootstrap randomization algorithms [41]. Probably the most commonly used are hierarchical clustering algorithms. For these spider data at the global level we opted to use the UPGMA with Sørensen similarity measure analysis, as these methods were already used for previous spider classifications [18].

Any dichotomous clustering method, such as UPGMA, divides taxa in 2 to n groups, with n equal to the number of taxa. The definition of guilds implies this division. However, it may be difficult to determine where the cut-off of each branch in the tree representing a separate guild should be made. We decided *a priori* to use a set of rules to achieve important practical goals. Firstly, enough guilds should be recognized to allow useful comparisons of sites based on guild proportions. Secondly, guilds should not be so numerous that the smallest would contain only one or very few families. Thirdly, the guilds should be as homogenous as possible, although some exceptions were inevitable, for example due to major divergent lifestyles inside a single family. Although these rules require some subjectivity, their application after quantitative analysis mitigated this weakness.

Finally, in order to verify that the defined guilds were statistically supported, we performed an analysis of similarity (ANOSIM). This statistic employs a randomization technique that compares the within and between group similarity of elements as measured by the Bray-Curtis index. Because we used presence/absence data, this index was equivalent to the Sørensen index, also used for building the tree, making both statistical approaches fully comparable.

### Sampling effort

Richness comparisons of assemblages must always be made cautiously; in particular sampling completeness should not differ. If some guilds were differentially sampled by the methods employed, different completeness values would compromise direct comparisons. We first calculated for each site each guild's estimated richness with the Chao1 estimator [48] and calculated completeness as the observed to estimated richness ratio. However, the Chao estimates were far from reliable, and the completeness variance of the different guilds belonging to each site was very large (results not shown). The completeness values were therefore unreliable. As an alternative to completeness, we estimated the final slopes of guild species richness accumulation curves for all guilds at each site. All curves were sample-based, randomized 1000 times and rescaled to individuals as suggested by Gotelli and Colwell [49]. The final slopes of curves were calculated as:
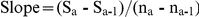
where S_a_ = total number of species; S_a-1_ = number of species after adding the next to last sample; n_a_ = total number of individuals; n_a-1_ = number of individuals after adding the next to last sample. The slopes at the end of the accumulation curves for all guilds did not differ significantly (Kruskal-Wallis test: H_7,64_ = 10.978; p = 0.140). Thus all guilds were sampled at a similar rate during the accumulation process such that higher sampling completeness at some sites did not influence the proportion of species richness per guild at each site.

### Higher taxa surrogacy

Our datasets included mainly tropical assemblages, for which a large part of the morphospecies could not be assigned to known species or even genera. Therefore, to test the higher taxa surrogacy hypothesis, we used only the two Portugal datasets (Table 1) for which substantial information about most species was available. For both datasets, which had intermediate family and species richness values, we calculated the number of species and individuals per guild as identified by the methods above. We then corrected guild placement using the knowledge we had on each species, and again calculated the number of species and individuals per guild. To test the surrogacy, we used a bootstrapping procedure to evaluate if the richness and abundance differed statistically for each guild. We also tested the correlation of richness and abundance per guild before and after correction with the Spearman rank statistic.

### Taxonomic and guild composition

To compare the taxonomic and guild composition between sites we compared the proportions of each guild and family between every possible pair of sites using the Spearman rank correlation index. If guilds have relatively similar proportions worldwide, their correlations should be higher than the respective family proportion correlations. For these analyses we used only the eight most rich or abundant families on average for all sites, so that rare families did not artificially decrease rank correlation values of the family comparisons.

### Functional diversity

Although many options exist for calculating functional diversity (FD) [15], [50], none are optimal in all cases [51]. For consistency we used the same UPGMA tree used to define guilds. FD was calculated as the sum of lengths of the branches connecting all families observed in a particular site [16]. The complete tree with all 108 spider families was used for all sites.

Functional diversity depends strongly on taxa richness [16], because more taxa imply more branches in the tree. Richness, in turn, strongly depends on the number of observed individuals. We therefore resampled the data for each site by randomly selecting 1000 individuals and calculating the FD value of the resulting tree. This resampling was made 1000 times per site, allowing obtaining 95% confidence limits calculated as the respective 0.025 and 0.975 percentiles. All calculations were made with Java software written specifically for this work (available from the first author by request).

## Results

### Definition of guilds

**Figure 1:**
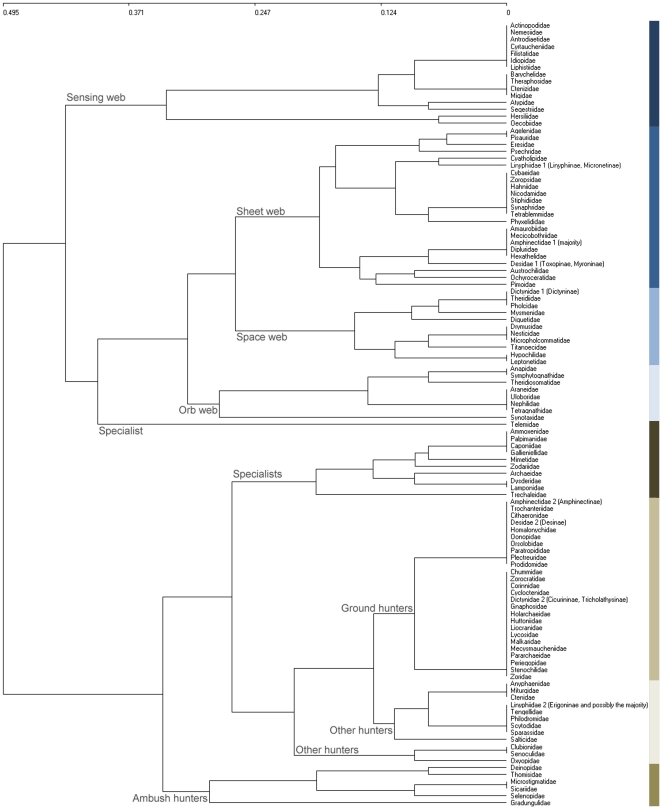
Hierarchical clustering of families. Results for the UPGMA analysis with Sørensen index of dissimilarity applied to the ecological characteristics of spider families. Names of eight distinguished guilds are shown.

As mentioned above, we recognized subgroups within four families (Amphinectidae, Desidae, Dictynidae and Linyphiidae) because their subfamilies exhibited disparate strategies, and treated these as equal to families. A total of eight guilds could be discriminated from the UPGMA analysis (Fig. 1, Table S1): (1) sensing web weavers, (2) sheet web weavers, (3) space web weavers, (4) orb web weavers, (5) specialists, (6) ambush hunters, (7) ground hunters and (8) other hunters. The ANOSIM analyses supported the recognized guilds (global R = 0.917, p<0.001; 0.695<R<1, p<0.001 in all cases). Guilds ranged in size from relatively large (ground hunters, 26 families) to small (ambush hunters, six families). As these separated from other families relatively deeply in the tree, their discrimination seemed justified.

### Higher taxa surrogacy

Out of 173 species and 3268 specimens in the Portuguese datasets, 27 species (16%) and 358 specimens (11%) were incorrectly attributed to a guild by family surrogacy. These included stenophagous species that belong to families where stenophagy is not very common (e.g., the theridiid ant specialists *Dipoena* and *Euryopis*, the araneophagic jumping spider *Cyrba*, the gnaphosid ant specialists *Callilepis* and *Nomisia*) or generalist species in specialist families (e.g., *Harpactea* and *Rhode* in Dysderidae). Also, some higher stratum species occur in typically ground hunting families (e.g., *Echemus* and *Scotophaeus* in Gnaphosidae), and hunting species occur in some web-building families (e.g. *Pisaura* in Pisauridae), etc.

**Figure 2:**
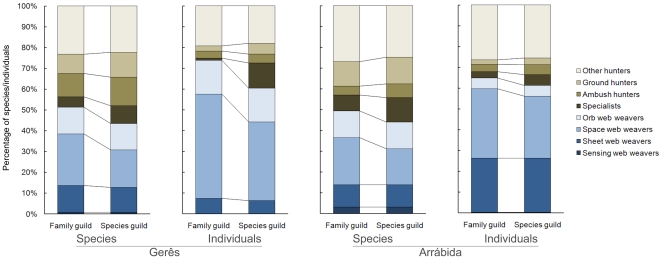
Guild richness and abundance per site with family and species guild classifications. Percentage of species and individuals in each of two sites in Portugal belonging to each guild according to the respective family and species classifications (family classification reflects the predominant guild in the family (Fig. 1), while species classification represents the true guild of the species).

Bootstrapping confirmed these differences in numbers of species and individuals per guild, with space web weavers presenting lower values and specialists higher values after correcting species guild placement (Fig. 2). Statistically significant differences were also found for ambush hunters' richness and ground hunters' abundance in both sites, as well as ground hunters' richness in Gerês and ambush hunters' abundance in Arrábida.

However, even given significant differences in richness and abundance of some guilds before and after correction, the Spearman rank correlation of uncorrected vs. corrected guilds was invariably high (0.833<R<0.970; n = 8; 0.00007<p<0.01 in all cases).

### Taxonomic and guild composition

**Figure 3:**
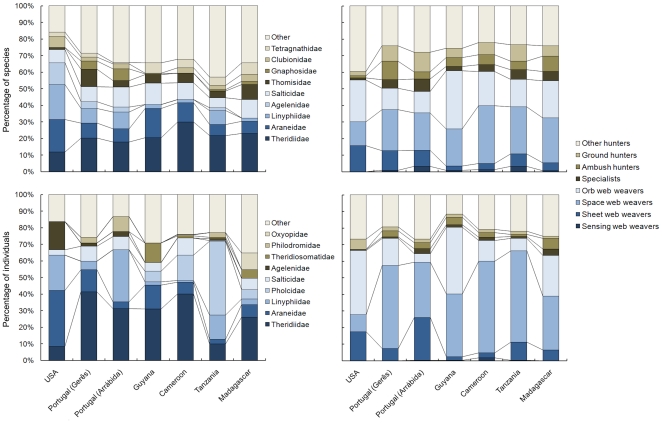
Percentage of species and individuals per family and guild in each studied site.

In four cases, the space weavers were the richest guild (between 24 and 35% of species; Fig. 3). In Guyana orb weavers were the richest, at 35% of species. In USA and Portugal (Arrábida), the “other hunters” guild was the richest with 39% and 28% of species respectively. Theridiids were richest in six sites, with 12 to 29% of species (Fig. 2). The exception occurred in the USA where Linyphiidae (21%) and Araneidae (20%) dominated.

In all cases except the USA and Guyana the most abundant guilds were the space web weavers, with 33 to 55% of the individuals captured (Fig. 3). In both exceptions the orb weavers were most abundant (39 and 40% respectively). Theridiidae was most abundant in five sites, with 26 to 41% of the individuals (Fig. 3). However, in the USA Theridiidae was only 9% of the total abundance, with Araneidae (34%), Linyphiidae (21%) and Agelenidae (17%) being more abundant. In Tanzania, the Pholcidae were most abundant (44%), followed by Linyphiidae (15%), and Theridiidae (10%).

In 19 out of 21 pairwise comparisons of sites (90%) the correlation of guild species richness was higher than the correlation of family species richness (Table 2). Significant correlations were found for guilds in 19 cases (90%), but only in 11 cases (52%) for families. In 20 out of 21 pairwise comparisons of sites (95%) the correlation of guild abundance was higher than the correlation of family abundance (Table 2). Correlations were significant in 19 cases (90%) for guilds, but only in three cases (14%) for families.

**Table 2:** Pairwise Spearman rank correlations of family and guild richness and abundance of studied sites.

	USA	Portugal (Gerês)	Portugal (Arrábida)	Guyana	Cameroon	Tanzania	Madagascar
**Richness**							
USA	1	0.814*	0.874**	0.777*	0.635	0.802*	0.599
Portugal (Gerês)	0.122	1	0.857**	0.778*	0.833*	0.881**	0.857**
Portugal (Arrábida)	0.299	0.724*	1	0.850**	0.881**	0.976***	0.810*
Guyana	0.216	0.834**	0.801*	1	0.898**	0.826*	0.874**
Cameroon	0.180	0.700	0.633	0.946***	1	0.905**	0.976***
Tanzania	0.619	0.732*	0.778*	0.814*	0.755*	1	0.833*
Madagascar	−0.238	0.708*	0.611	0.850**	0.886**	0.524	1
**Abundance**							
USA	1	0.762*	0.690	0.762*	0.762*	0.762*	0.643
Portugal (Gerês)	0.725*	1	0.952***	0.905**	1.000***	0.952***	0.952***
Portugal (Arrábida)	0.691	0.852**	1	0.786*	0.952***	0.929***	0.929***
Guyana	0.123	0.515	0.158	1	0.905**	0.762*	0.905**
Cameroon	−0.122	0.439	0.193	0.802*	1	0.952***	0.952***
Tanzania	0.220	0.146	0.133	0.479	0.500	1	0.833*
Madagascar	−0.146	0.390	0.205	0.479	0.548	−0.143	1

Family below and guild above diagonals. For family richness and abundance we only used the eight most rich or abundant families on average for all sites.

*p<0.05;

**p<0.01;

***p<0.001.

### Functional diversity

**Figure 4:**
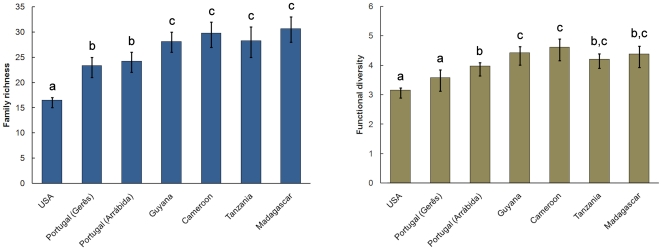
Average family richness and functional diversity per site (after resampling for 1000 individuals). Letters represent statistically different (at α<0.05) groups as determined by the resampling procedure.

Family richness was higher in the tropics than in temperate regions, and the Mediterranean sites in Portugal were intermediate (Fig. 4). Functional diversity presented a different pattern. The USA site and the northernmost site in Portugal (Gerês) had the lowest FD, but the more southern Portuguese site (Arrábida) and some tropical sites had similar values (Fig. 4). Families in less rich sites apparently filled the functional tree almost as completely as families in richer sites, similarly absorbing the available resource space.

## Discussion

Separating species into guilds can be as simple as grouping taxa according to trophic level [6]; or as complex as studying all the relationships between taxa in a multidimensional matrix [43]. The approach taken depends on: (1) the objectives of the study (the level of detail depends on the information needed to answer particular questions); (2) the spatial scale of the study (a worldwide guild classification aims at more general patterns than those at the assemblage level); (3) the taxonomic scale (classifying species requires different data than genera or families, whose species may perform different roles in the same assemblage); (4) the data reasonably available (quantitative data about all taxa and interactions is feasible only for single communities). As we attempted to characterize a megadiverse group at a global scale, our approach was designed to test questions at this level. In any case, most tropical spider species are undescribed and discriminated only as morphospecies. Moreover, ecological characteristics (foraging strategy, prey range, vertical stratification and circadian activity) for those species which are identified at specific or generic levels, are usually unknown. In such cases, family or even genus characteristics are available to aid in guild assignment. At a global level for spiders, families, and occasionally groups within families, are the most practical basis for guild classification.

As expected, the most important characteristic for defining guild placement was foraging strategy. Web type or hunting methods primarily determine the division of spider families into guilds, as was previously recognized by different authors dealing with this taxon [18], [19]. Hunting spiders could be further divided according to the vertical stratum preferred; stenophagous families constituted a separate guild. The eight guilds now proposed are partly coincident with previous classifications dealing with fewer families in smaller areas. Uetz and colleagues [18], studying crops in the USA, also proposed eight guilds. These authors, however, included Linyphiids in their own wandering sheet/tangle weavers guild and many families that we denominate as “other hunters” were considered as either foliage runners or stalkers. Dias and colleagues [19], studying Neotropical spiders, further refined many of the guilds in diurnal and nocturnal. In our global study, circadian activity was not decisive for guild placement. Circadian activity, along with phenology and body size, could however be used in smaller-scale or species-based studies. Species hunting in different times of day or seasons or having different body sizes probably are not sharing resources.

No previous study considered the problem of stenophagy and specialization of prey. It may be important to recognize in guild placement that specialist taxa have little overlap in resource sharing with other species. In that sense, the specialists' guild is not even a true guild, but a cluster of species that, by specializing in one or very few prey, are not directly competing with any large group of species.

Some families may seem to be at odds with the common perception of where they belong in the tree. This may result from lack of knowledge of the biology of tropical as compared to temperate species, or that different species have indeed developed different hunting strategies. For example, scytodids are usually regarded as ground hunters in the Holarctic, but they predominantly hunt on vegetation in the tropics. Deinopids are specialized orb weavers whose web is modified to ambush prey. We therefore characterize deinopids as ambush predators rather than as typical orb weavers.

In this work we assumed that guilds are largely conserved within families and that this conservatism was valid at a global level. The first assumption was tested with the Portugal datasets; our results supported the use of families as a surrogate for species guild classification. We could not test the second assumption because many individuals are not identified to species or genera in most tropical datasets. The biology of named tropical species moreover is usually unknown. However, current knowledge generally indicates that confamilial spider species tend to have the same lifestyle [23]. This tendency supports the application of these findings and guild classification to global biomes and habitats, although the validity of family surrogacy should be tested whenever possible to confirm the results here obtained. Even where direct knowledge of species permits a refined classification of guilds in a particular study, using a standard set of guilds and guild denominations will facilitate comparison of studies performed by different teams with different objectives as long as the methodology used to obtain data is comparable [34].

Despite the apparent support for the use of family guilds as surrogates for species guilds, exceptions are obvious. The difference in Gerês in the abundance of specialists between family and species-level guilds is mainly due to the high abundance of *Dipoena melanogaster* (C.L. Koch, 1837), the most commonly sampled species [30]. Although this theridiid was assigned to the space web builders' guild according to its family, *D. melanogaster* is a webless stenophagous ant specialist. At the global level, the bolas spiders (genera *Cladomelea*, *Mastophora* and *Ordgarius*) are also exceptions because these specialized hunters construct “bolas” that depend on aggressive chemical mimicry of a few moth species rather than typical orb webs [52]. The triangular araneid spiders *Arkys* have abandoned web building altogether and ambush prey with large front legs like those of thomisids. Cybaeidae usually build sheet-webs, but the water spider *Argyroneta aquatica* (Clerck, 1757) builds a silk retreat under water where it hunts without using a web during the entire life cycle. Although many pisaurids are active hunters, most spin large webs, while *Dolomedes* hunts on the water surface, occasionally for small fish (in fact, the monophyly of all Pisauridae is questionable). The same pattern repeats within smaller lineages recognized as genera. *Anapistula ataecina* Cardoso & Scharff, 2009 spin sheet-webs, not typical *Anapistula* orbs, probably because of their subterranean habitat [53]. Even with many exceptions, members of the same family do tend to present similar ecological characteristics; hence taxonomical affiliation often is associated with guild affiliation and the high correlation values found by this study. The guilds suggested here can be applied with care in many studies at various geographic scales.

Proportions of guilds per site necessarily depend on the sampling methods used. Most of our study datasets lacked data for pitfall traps, even though the method often captures more epigean fauna than any other method [30], [31], [33], [37], and therefore we could not properly assess ground hunters across datasets. The precise proportions per guild (and family) found are therefore specific to the methods available for comparison.

As predicted, guild composition was more stable than taxonomic composition, suggesting turnover in families using similar resources in different regions. Pholcids are relatively rare in the temperate and Mediterranean faunas, but common throughout the tropics and even dominant in the Tanzanian site. Linyphiids are much more abundant in temperate regions than elsewhere in the subtropics and tropics (except Tanzania, where they are atypically diverse). Other families show this pattern of high abundance in some regions but relative rarity in other regions (e.g. Salticidae, Agelenidae, Theridiosomatidae, Philodromidae and Oxyopidae).

Higher family richness in the tropics but similar functional diversity suggest either greater functional role redundancy compared to temperate forests or finer resource partitioning. In a community with higher redundancy, the role of any one taxon may be at least partly compensated by another taxon as the niches of syntopic taxa present higher overlap and therefore it is easier for the assemblage to keep its structure under disturbance and harder for invading species to occupy “empty” niches. High ecological redundancy may underlie the resilience of ecosystems to disturbance and invasive species [40], [54]–[56]. Assemblages with particularly low functional diversity and very simple food chains, such as sites in extreme altitudes or latitudes, small isolated oceanic islands and caves, may be more susceptive to disruption [57]. Compared to other assemblages, these four examples may present: 1) low redundancy and therefore greater susceptibility to disturbance; and 2) no suitable refuge from such disturbance. Such assemblages are thus particularly vulnerable to habitat destruction, invasive species and climate change. This diversity-stability relation may explain why oceanic island assemblages, with few species and sparse guilds, are especially prone to extinctions, including for spiders [17]. The patterns found in this work may also be partly explained by finer resource partitioning instead of or besides redundancy. In the tropics many species can present higher specialization in response to higher competition from syntopic taxa. This results in narrower niches and higher number of species or higher taxa per guild.

Functional diversity in Tanzania and Madagascar was less than in other tropical sites and not statistically different from the Portugal (Arrábida) site. These two tropical sites are however at mid to high altitude. Comparing the three sites in temperate/Mediterranean regions, at similar latitudes, the same tendency is present, with the mid-altitude sites presenting a lower functional diversity than the low-altitude site. If altitude correlates to some extent with habitat complexity, this may have caused overall functional diversity to be lower. As noted, this study is an analysis of datasets less than ideal in numerous ways for the present purpose. However, given the study's drawbacks, we find some evidence that functional diversity does decrease with overall habitat complexity, whether due to high elevations or high latitudes.

In conclusion, we have, for the first time, proposed a global classification of spider guilds including every extant family. Our results suggest that families may be statistically adequate ecological surrogates for species, thus providing a consistent framework for future developments in the area, although the validity of surrogacy should be further tested in different areas. Even if adjustments have to be made in some regions, with some species moving between guilds, a consistent guild classification can promote future comparison between different geographic regions and habitats. We also suggest that different families may have similar ecological roles, with replacement of some taxa by other within the same guild according to the region. Guild structure may therefore be predictable and independent of taxonomic structure. Our work also indicates that tropical regions may have higher redundancy of functional roles and/or finer resource partitioning than temperate regions. If the diversity-stability relation is confirmed, this may be an indication of higher resistance to disturbance in high-diversity, tropical forests than in low-diversity, temperate forests. Finally, functional diversity may correlate with habitat structure and seems to be higher in low elevation forests, possibly with higher vegetation complexity.

## Supporting Information

Table S1 **List of spider families with respective ecological characteristics and the resulting guild category.**
(PDF)
