# Supplementary material for: Global Patterns of Guild Composition and Functional Diversity of Spiders
Source: PLoS One. 2011 Jun 29;6(6):e21710. doi: 10.1371/journal.pone.0021710 (PMC3126856; doi:10.1371/journal.pone.0021710)
Supplement: Table S1 — List of spider families with respective ecological characteristics and the resulting guild category. (PDF) [file pone.0021710.s001.pdf]

Table S1. List of spider families with respective ecological characteristics and the resulting guild category.

Family	Capture web	Sensing web	No web	Tube web	Sheet web	Space web	Orb web	Ambush hunter	Active hunter	Stenophagous	Euryphagous	Ground	Vegetation	Diurnal	Nocturnal	Guild
**Actinopodidae**	0	1	0	1	0	0	0	0	0	0	1	1	0	0	1	Sensing web weavers
**Agelenidae**	1	0	0	1	1	0	0	0	0	0	1	1	1	1	1	Sheet web weavers
**Amaurobiidae**	1	0	0	1	1	0	0	0	0	0	1	1	0	0	1	Sheet web weavers
**Ammoxenidae**	0	0	1	0	0	0	0	0	1	1	0	1	0	1	1	Specialists
**Amphinectidae 1 (majority)**	1	0	0	1	1	0	0	0	0	0	1	1	0	0	1	Sheet web weavers
**Amphinectidae 2 (Amphinectinae)**	0	0	1	0	0	0	0	0	1	0	1	1	0	0	1	Ground hunters
**Anapidae**	1	0	0	0	0	0	1	0	0	0	1	1	0	1	1	Orb web weavers
**Antrodiaetidae**	0	1	0	1	0	0	0	0	0	0	1	1	0	0	1	Sensing web weavers
**Anyphaenidae**	0	0	1	0	0	0	0	0	1	0	1	1	1	0	1	Other hunters
**Araneidae**	1	0	0	0	0	0	1	0	0	0	1	0	1	1	1	Orb web weavers
**Archaeidae**	0	0	1	0	0	0	0	0	1	1	0	1	1	0	1	Specialists
**Atypidae**	0	1	0	1	0	0	0	0	0	0	1	1	0	1	1	Sensing web weavers
**Austrochilidae**	1	0	0	0	1	0	0	0	0	0	1	1	0	0	1	Sheet web weavers
**Barychelidae**	0	1	0	1	0	0	0	0	0	0	1	1	1	0	1	Sensing web weavers
**Caponiidae**	0	0	1	0	0	0	0	0	1	1	0	1	0	1	1	Specialists
**Chummidae**	0	0	1	0	0	0	0	0	1	0	1	1	0	1	1	Ground hunters
**Cithaeronidae**	0	0	1	0	0	0	0	0	1	0	1	1	0	0	1	Ground hunters
**Clubionidae**	0	0	1	0	0	0	0	0	1	0	1	0	1	0	1	Other hunters
**Corinnidae**	0	0	1	0	0	0	0	0	1	0	1	1	0	1	1	Ground hunters
**Ctenidae**	0	0	1	0	0	0	0	0	1	0	1	1	1	0	1	Other hunters
**Ctenizidae**	0	1	0	1	0	0	0	0	0	0	1	1	1	0	1	Sensing web weavers
**Cyatholipidae**	1	0	0	0	1	0	0	0	0	0	1	1	1	1	1	Sheet web weavers
**Cybaeidae**	1	0	0	0	1	0	0	0	0	0	1	1	0	1	1	Sheet web weavers
**Cycloctenidae**	0	0	1	0	0	0	0	0	1	0	1	1	0	1	1	Ground hunters
**Cyrtaucheniidae**	0	1	0	1	0	0	0	0	0	0	1	1	0	0	1	Sensing web weavers
**Deinopidae**	0	0	1	0	0	0	0	1	0	0	1	0	1	1	1	Ambush hunters
**Desidae 1 (Toxopinae, Myroninae)**	1	0	0	1	1	0	0	0	0	0	1	1	1	0	1	Sheet web weavers
**Desidae 2 (Desinae)**	0	0	1	0	0	0	0	0	1	0	1	1	0	0	1	Ground hunters
**Dictynidae 1 (Dictyninae)**	1	0	0	0	0	1	0	0	0	0	1	1	1	1	1	Space web weavers
**Dictynidae 2 (Cicurininae, Tricholathysinae)**	0	0	1	0	0	0	0	0	1	0	1	1	0	1	1	Ground hunters
**Diguetidae**	1	0	0	0	0	1	0	0	0	0	1	1	1	1	0	Space web weavers
**Dipluridae**	1	0	0	1	1	0	0	0	0	0	1	1	0	0	1	Sheet web weavers
**Drymusidae**	1	0	0	0	0	1	0	0	0	0	1	1	0	1	1	Space web weavers
**Dysderidae**	0	0	1	0	0	0	0	0	1	1	0	1	0	0	1	Specialists
**Eresidae**	1	0	0	1	1	1	0	0	0	0	1	1	1	1	1	Sheet web weavers
**Filistatidae**	0	1	0	1	0	0	0	0	0	0	1	1	0	0	1	Sensing web weavers
**Gallieniellidae**	0	0	1	0	0	0	0	0	1	1	0	1	0	1	1	Specialists
**Gnaphosidae**	0	0	1	0	0	0	0	0	1	0	1	1	0	1	1	Ground hunters
**Gradungulidae**	0	0	1	0	0	0	0	1	0	1	0	1	0	0	1	Ambush hunters
**Hahniidae**	1	0	0	0	1	0	0	0	0	0	1	1	0	1	1	Sheet web weavers
**Hersiliidae**	0	1	0	0	1	0	0	1	0	0	1	1	1	1	1	Sensing web weavers
**Hexathelidae**	1	0	0	1	1	0	0	0	0	0	1	1	0	0	1	Sheet web weavers

**Holarchaeidae**	0	0	1	0	0	0	0	0	1	0	1	1	0	1	1	Ground hunters
**Homalonychidae**	0	0	1	0	0	0	0	0	1	0	1	1	0	0	1	Ground hunters
**Huttoniidae**	0	0	1	0	0	0	0	0	1	0	1	1	0	1	1	Ground hunters
**Hypochilidae**	1	0	0	0	0	1	0	0	0	0	1	1	0	0	1	Space web weavers
**Idiopidae**	0	1	0	1	0	0	0	0	0	0	1	1	0	0	1	Sensing web weavers
**Lamponidae**	0	0	1	0	0	0	0	0	1	1	0	1	0	0	1	Specialists
**Leptonetidae**	1	0	0	0	0	1	0	0	0	0	1	1	0	0	1	Space web weavers
**Linyphiidae 1 (Linyphiinae, Micronetinae)**	1	0	0	0	1	1	0	0	0	0	1	1	1	1	1	Sheet web weavers
**Linyphiidae 2 (Erigoninae and others)**	0	0	1	0	0	0	0	0	1	0	1	1	1	1	1	Other hunters
**Liocranidae**	0	0	1	0	0	0	0	0	1	0	1	1	0	1	1	Ground hunters
**Liphistiidae**	0	1	0	1	0	0	0	0	0	0	1	1	0	0	1	Sensing web weavers
**Lycosidae**	0	0	1	0	0	0	0	0	1	0	1	1	0	1	1	Ground hunters
**Malkaridae**	0	0	1	0	0	0	0	0	1	0	1	1	0	1	1	Ground hunters
**Mecicobothriidae**	1	0	0	1	1	0	0	0	0	0	1	1	0	0	1	Sheet web weavers
**Mecysmaucheniidae**	0	0	1	0	0	0	0	0	1	0	1	1	0	1	1	Ground hunters
**Micropholcommatidae**	1	0	0	0	0	1	0	0	0	0	1	1	0	1	1	Space web weavers
**Microstigmatidae**	0	0	1	0	0	0	0	1	0	0	1	1	0	0	1	Ambush hunters
**Migidae**	0	1	0	1	0	0	0	0	0	0	1	1	1	0	1	Sensing web weavers
**Mimetidae**	0	0	1	0	0	0	0	0	1	1	0	1	1	1	1	Specialists
**Miturgidae**	0	0	1	0	0	0	0	0	1	0	1	1	1	0	1	Other hunters
**Mysmenidae**	1	0	0	0	0	1	1	0	0	0	1	1	1	1	1	Space web weavers
**Nemesiidae**	0	1	0	1	0	0	0	0	0	0	1	1	0	0	1	Sensing web weavers
**Nephilidae**	1	0	0	0	0	0	1	0	0	0	1	0	1	1	1	Orb web weavers
**Nesticidae**	1	0	0	0	0	1	0	0	0	0	1	1	0	1	1	Space web weavers
**Nicodamidae**	1	0	0	0	1	0	0	0	0	0	1	1	0	1	1	Sheet web weavers
**Ochyroceratidae**	1	0	0	0	1	1	0	0	0	0	1	1	0	0	1	Sheet web weavers
**Oecobiidae**	0	1	0	0	1	0	0	1	0	0	1	1	0	1	1	Sensing web weavers
**Oonopidae**	0	0	1	0	0	0	0	0	1	0	1	1	0	0	1	Ground hunters
**Orsolobidae**	0	0	1	0	0	0	0	0	1	0	1	1	0	0	1	Ground hunters
**Oxyopidae**	0	0	1	0	0	0	0	0	1	0	1	0	1	1	1	Other hunters
**Palpimanidae**	0	0	1	0	0	0	0	0	1	1	0	1	0	1	1	Specialists
**Pararchaeidae**	0	0	1	0	0	0	0	0	1	0	1	1	0	1	1	Ground hunters
**Paratropididae**	0	0	1	0	0	0	0	0	1	0	1	1	0	0	1	Ground hunters
**Periegopidae**	0	0	1	0	0	0	0	0	1	0	1	1	0	1	1	Ground hunters
**Philodromidae**	0	0	1	0	0	0	0	0	1	0	1	1	1	1	1	Other hunters
**Pholcidae**	1	0	0	0	0	1	0	0	0	0	1	1	1	1	1	Space web weavers
**Phyxelididae**	1	0	0	0	1	1	0	0	0	0	1	1	0	1	1	Sheet web weavers
**Pimoidae**	1	0	0	0	1	0	0	0	0	0	1	1	1	0	1	Sheet web weavers
**Pisauridae**	1	0	0	1	1	0	0	0	0	0	1	1	1	1	1	Sheet web weavers
**Plectreuridae**	0	0	1	0	0	0	0	0	1	0	1	1	0	0	1	Ground hunters
**Prodidomidae**	0	0	1	0	0	0	0	0	1	0	1	1	0	0	1	Ground hunters
**Psechridae**	1	0	0	1	1	0	0	0	0	0	1	0	1	1	1	Sheet web weavers
**Salticidae**	0	0	1	0	0	0	0	0	1	0	1	1	1	1	0	Other hunters
**Scytodidae**	0	0	1	0	0	0	0	0	1	0	1	1	1	1	1	Other hunters
**Segestriidae**	0	1	0	1	0	0	0	0	0	0	1	1	1	1	1	Sensing web weavers
**Selenopidae**	0	0	1	0	0	0	0	1	0	0	1	1	1	0	1	Ambush hunters
**Senoculidae**	0	0	1	0	0	0	0	0	1	0	1	0	1	0	1	Other hunters
**Sicariidae**	0	0	1	0	0	0	0	1	0	0	1	1	0	0	1	Ambush hunters
**Sparassidae**	0	0	1	0	0	0	0	0	1	0	1	1	1	1	1	Other hunters
**Stenochilidae**	0	0	1	0	0	0	0	0	1	0	1	1	0	1	1	Ground hunters
**Stiphidiidae**	1	0	0	0	1	0	0	0	0	0	1	1	0	1	1	Sheet web weavers
**Symphytognathidae**	1	0	0	0	0	0	1	0	0	0	1	1	0	1	1	Orb web weavers

**Synaphridae**	1	0	0	0	1	0	0	0	0	0	1	1	0	1	1	Sheet web weavers
**Synotaxidae**	1	0	0	0	1	1	1	0	0	0	1	0	1	0	1	Orb web weavers
**Telemidae**	1	0	0	0	1	0	0	0	0	1	0	1	0	0	1	Specialists
**Tengellidae**	0	0	1	0	0	0	0	0	1	0	1	1	1	1	1	Other hunters
**Tetrablemmidae**	1	0	0	0	1	0	0	0	0	0	1	1	0	1	1	Sheet web weavers
**Tetragnathidae**	1	0	0	0	0	0	1	0	0	0	1	0	1	1	1	Orb web weavers
**Theraphosidae**	0	1	0	1	0	0	0	0	0	0	1	1	1	0	1	Sensing web weavers
**Theridiidae**	1	0	0	0	0	1	0	0	0	0	1	1	1	1	1	Space web weavers
**Theridiosomatidae**	1	0	0	0	0	0	1	0	0	0	1	1	1	1	1	Orb web weavers
**Thomisidae**	0	0	1	0	0	0	0	1	0	0	1	1	1	1	1	Ambush hunters
**Titanoecidae**	1	0	0	1	0	1	0	0	0	0	1	1	0	1	1	Space web weavers
**Trechaleidae**	0	0	1	0	0	0	0	0	1	1	0	0	1	1	1	Specialists
**Trochanteriidae**	0	0	1	0	0	0	0	0	1	0	1	1	0	0	1	Ground hunters
**Uloboridae**	1	0	0	0	0	0	1	0	0	0	1	0	1	1	1	Orb web weavers
**Zodariidae**	0	0	1	0	0	0	0	1	1	1	0	1	0	1	1	Specialists
**Zoridae**	0	0	1	0	0	0	0	0	1	0	1	1	0	1	1	Ground hunters
**Zorocratidae**	0	0	1	0	0	0	0	0	1	0	1	1	0	1	1	Ground hunters
**Zoropsidae**	1	0	0	0	1	0	0	0	0	0	1	1	0	1	1	Sheet web weavers
